# COEUS: “semantic web in a box” for biomedical applications

**DOI:** 10.1186/2041-1480-3-11

**Published:** 2012-12-17

**Authors:** Pedro Lopes, José Luís Oliveira

**Affiliations:** 1 DETI/IEETA, Universidade de Aveiro, Campus Universitário de Santiago, Aveiro, 3810 – 193, Portugal

**Keywords:** Semantic web framework, Rapid application deployment, Linked data, Web services, Biomedical applications, Biomedical semantics

## Abstract

**Background:**

As the “omics” revolution unfolds, the growth in data quantity and diversity is bringing about the need for pioneering bioinformatics software, capable of significantly improving the research workflow. To cope with these computer science demands, biomedical software engineers are adopting emerging semantic web technologies that better suit the life sciences domain. The latter’s complex relationships are easily mapped into semantic web graphs, enabling a superior understanding of collected knowledge. Despite increased awareness of semantic web technologies in bioinformatics, their use is still limited.

**Results:**

COEUS is a new semantic web framework, aiming at a streamlined application development cycle and following a “semantic web in a box” approach. The framework provides a single package including advanced data integration and triplification tools, base ontologies, a web-oriented engine and a flexible exploration API. Resources can be integrated from heterogeneous sources, including CSV and XML files or SQL and SPARQL query results, and mapped directly to one or more ontologies. Advanced interoperability features include REST services, a SPARQL endpoint and LinkedData publication. These enable the creation of multiple applications for web, desktop or mobile environments, and empower a new knowledge federation layer.

**Conclusions:**

The platform, targeted at biomedical application developers, provides a complete skeleton ready for rapid application deployment, enhancing the creation of new semantic information systems. COEUS is available as open source at http://bioinformatics.ua.pt/coeus/.

## Background

Emerging semantic web technologies are the perfect solution for life sciences challenges. In the last two decades we have witnessed an unprecedented explosion of data originated at novel software and hardware platforms, leveraging a prolific research community in constant demand of best-of-breed tools. Hence, this domain is evolving exponentially and reaching user profiles far beyond the traditional wet-lab biologist [1,2]. Innovative biomedical technologies span the entire life sciences spectrum, crossing miscellaneous areas of work, and culminating in a need for improved integrative software that fosters research interoperability. For example, the use of next-generation sequencing hardware, mapping human genetic sequences to digital data, has grown steadily, spawning enormous amounts of data in each read [3]. While it is essential to make sense of these data in order to realize the immense potential for custom drug development and personalized medicine, its understanding relies on expertise from various life sciences fields and connects researchers with distinct needs and expectations, from gene curators to pharmaceutical researchers and medical clinicians.

Despite the key role that bioinformatics software and hardware developments have played over the last years, the life sciences technological ecosystem is still fragmented and contains immeasurable entropy. The majority of data is scattered through closed independent systems, disregarding any good practice for integration and interoperability features [4]. Furthermore, even in notable state-of-the-art tools, the overwhelming scale and complexity of collected data and features generates an information overload, making it impossible for researchers to grasp deep insights from available knowledge [5,6].

Semantic web adoption within the life sciences community provided better paradigms, standards and technologies to solve common problems in biomedical sciences, such as data heterogeneity, diversity or distribution. Research from Slater *et al.*[7] and Kozhenkov *et al.*[8], among others, undermines current software development strategies, concluding that there is a clear need for new approaches adopting distinct ideals and based on a different set of skills. As next-generation sequencing technologies are breaking barriers in DNA sequencing, the semantic web may be seen as a next-generation software development paradigm, capable of breeding a new wave of more complete biomedical software solutions.

Taking into account the need for novel bioinformatics software with improved integration and interoperability features [9], the combination of semantic web technologies with innate life sciences characteristics will permit distinct computational systems to exchange and accurately interpret knowledge [10]. Semantic web technologies allow the development of systems that truly integrate and share information from clinical environments to diagnostic labs. Moreover, the semantic web itself is an intelligent data network, with rich connections allowing for a better understanding of available knowledge.

However, despite the immense possibilities surrounding semantic web technologies, their adoption has been slower than anticipated [11]. Whilst stakeholders from all domains acknowledge the benefits of having a fully semantic information system, the difficult transition from traditional flat-file or relational database systems to the semantic web is a challenging roadblock [12] and the subject of extensive research [13-16].

Evolving current applications to the semantic web ecosystem is a step that must be taken by software engineers in the coming years. With the tools currently available, this migration’s success is limited to a below-reasonable level. Moreover, developers must take into account what the needs of future software will be. If this is not considered, integration, interoperability, distribution and heterogeneity issues remain a problem. The combination of these factors with biomedical software requirements demands a new kind of application framework. Although the life sciences community is increasingly aware of semantic web’s advantages, it is imperative that novel software solutions streamline its development process, overcoming the semantic web’s steep learning curve and technological barrier.

With COEUS, we introduce a framework to tackle these challenges, empowering developers with a “semantic web in a box” software stack, and ensuring a more agile development workflow for new systems. The framework touches four issues currently found in the development of semantic web software: 1) the transition from primitive to semantically enhanced systems, 2) the integration and triplification of data, 3) the implementation of interoperability features and 4) the deployment of a transverse knowledge federation layer. The COEUS framework is available at http://bioinformatics.ua.pt/coeus/.

## Implementation

To better explain COEUS strategy we devised a naming strategy, following a gardening metaphor. A single COEUS instance is entitled as Knowledge Seed, or simply seed. In future scenarios with multiple seeds deployed in a true application ecosystem, this federated structure is envisaged as a Knowledge Garden. Next, we discuss the background for this transition from seeds to gardens, highlighting the requirements for integrating data and fostering interoperability through the exploration of acquired data.

### Model

**Figure 1:**
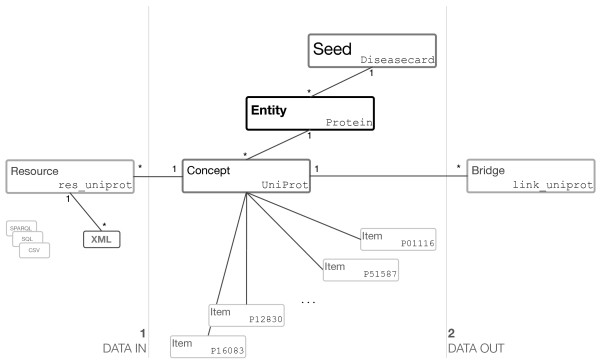
**COEUS ontology model for the internal tree-based structure highlighting relationships amongst the various individual classes.** A seed can have multiple entities, and each entity can be related to one or more concepts. Concepts aggregate unique items and are connected to resource and bridge information. The data import process uses resources’ properties (**1**) and custom methods can be defined to display data (**2**). Sample seed data for a “Diseasecard” seed is shown at each element, listing “UniProt” items belonging to a “Protein” entity.

To achieve the desired COEUS’ scalability and flexibility, the basic platform model is organized in a tree-based structure. Data relationships are mapped to Entity-Concept-Item structures, which are connected to Resources and Bridges, supporting integration and exploration settings, respectively (Figure 1).

To better understand this organization, object-oriented structures, their inheritance and variable subtypes must be remembered. This ontology is available online at http://bioinformatics.ua.pt/coeus/ontology/ and must be adopted in COEUS’ setup files. A short description for each core class follows.

 • A Seed defines a single framework instance. In COEUS’ model, Seed individuals are used to store a variety of application settings, such as component information, application descriptions, versioning or authors. Seed individuals are also connected to included entities through the :includes property (inverse of :isIncludedIn). This permits access to all data available in the seed, providing an over-arching entry point to the system information.

 • Entity individuals match the general data terms. These are “umbrella” elements, grouping concepts with a common set of properties (the :isEntityOf predicate).

 • Concept individuals are area-specific terms, aggregating any number of items (the :isConceptOf object property) and belonging to a unique entity (the :hasEntity object property).

 • Item individuals are the basic terms, with no further granularity and representing unique identifiers from integrated datasets. Item individuals are connected through the :isAssociatedTo object property and linked uniquely to a concept through the :hasConcept predicate.

 • Resource individuals are used to store external resource integration properties. The configuration is further specialized with CSV, XML, SQL and SPARQL classes, mapping precise dataset results to the application model, through direct concept relationships. With the :hasResource property, the framework knows exactly what resources are connected to each concept and, subsequently, how to load data for each independent concept, generating new items.

 • Brigde individuals are also mapped to concepts, storing concept visualization and exploration features. That is, bridges tell the system how concept items can be shown to users. This configuration permits any number of internal properties as long as they are understood by the final client application. This means that we can include parameters for advanced data visualizations, triggering web service calls or composing simple links.

Considering a proteomics scenario, the model has a “Protein” entity for aggregating protein information and a “Disease” entity for disease information. We can have sources such as “UniProt”, “PDB” and “InterPro” as concepts within the “Protein” entity. Below these, “P51587”, “P02461” are items under the “UniProt” concept, each matching a unique entry from the original UniProt database. For the “Disease” entity, the “104300” individual is a match for Alzheimer’s disease entry in the “OMIM” database concept.

To integrate UniProt data, a Resource individual contains information for the “UniProt” concept original source, including its location and how to extract each item. This resource is connected to several XML individuals (predicate :loadsFrom), each containing an XPath query whose results will map to application model properties. A bridge to display links to the original “UniProt” data source declares a structure for building valid UniProt URLs, replacing *#replace#* in http://www.uniprot.org/uniprot/#replace# with individual item identifiers.

One of the great advantages of using semantic web technologies is that any external ontology can be used to complement or extend COEUS internal model. As long as new predicates are understood by seed applications, any number of properties can be included, semantically mapping concepts or entities to existing ontologies, or adding further properties to describe entities, concepts, resources or bridges.

### Architecture

**Figure 2:**
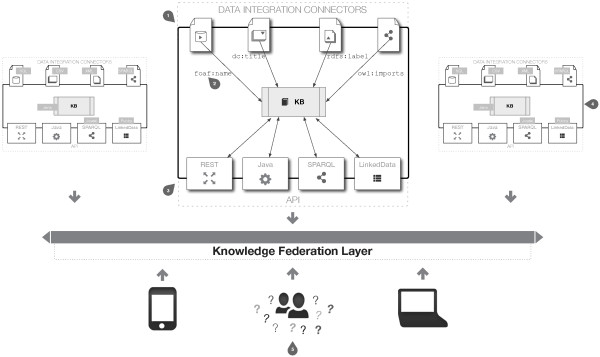
**COEUS architecture overview.** Data integration connectors for CSV, XML, SQL and SPARQL enable the triplification of data into each seed’s semantic storage (**1**). Data are selected from each external resource to match specific ontology predicates, generating a rich knowledge base (**2**). Acquired data are available through COEUS' API, using Java methods, REST services, a SPARQL endpoint and the LinkedData view (**3**). The SPARQL endpoint enables the connection of multiple knowledge seeds through a transparent knowledge federation layer (**4**). With one or more seeds in place, the COEUS platform enables answering specific research questions or deploying multiple applications to web, desktop or mobile environments (**5**).

COEUS allows for two main strategies to develop intelligent life sciences ecosystems, which can be viewed as a simple choice between “one to many” or “many to one”. On the one hand, a seed can accommodate multiple end-user applications, on distinct devices for instance. This means that a single centralized data source can be built to support any number of web, desktop or mobile applications. On the other hand, multiple specialized seeds can be connected to supply a single holistic application. In this strategy all seeds work independently, and can be seen as nodes in a data source network, providing access to an over-arching tool. Future scenarios may involve hybrid solutions, with a “many to many” architecture, combining multiple seeds in a distributed data and application ecosystem. Figure 2 highlights COEUS federation architecture, with multiple standalone seeds interconnected in a virtual knowledge network.

By default, a COEUS knowledge seed includes the necessary components to build and launch a new application quickly. In addition, developers just need a Java application server and a relational database (for the triplestore backend). COEUS is built on top of Jena (http://jena.apache.org/), whose Java-based nature, easy integration with other tools and extensibility, make it ideal for use in a component-based framework. Jena's API has basic support for reading and writing triple statements in Java. Since Joseki (http://www.joseki.org/) and Jena share common ancestry, the former was chosen as the default SPARQL server, being directly connected to the seed storage. Pubby (http://www4.wiwiss.fu-berlin.de/pubby/) is also included in COEUS package to facilitate the publication of data in the knowledge base as LinkedData. Finally, each seed includes a set of comprehensive data integration wrappers, entitled connectors, discussed further in this manuscript.

### Configuring seeds

A seed’s configuration controls the entire instance operability. For this matter, three separate files are used to define the application properties, the application model and resource integration setup. An in-depth description of these files is included in Table 1.

**Table 1:** COEUS seed configuration files purpose and description

**File Name**	**Purpose**	**Description**
config.js	Store volatile application properties.	This file configures the application name, version, short description, deployment environment and the list of ontologies used in the seed. Using a JSON object for the configuration permits faster reads when compared to XML, while maintaining a good object-oriented structure in comparison to simple properties files.
coeus_ontology.owl	Define the internal information system model.	In scenarios where the “reuse instead of rewrite” principle does not suffice for the entire application ecosystem, COEUS allows the creation of custom ontologies to use in one or various seeds. Developers are able to organize their own application models, taking full advantage of RDF/OWL’s modeling flexibility.
coeus_setup.rdf	Application setup file for integration and exploration details.	This file defines entities, concepts, bridges and resources. Summarily, the content is used to guide the entire framework instance setup, from the handling of external resources in the connectors to the labeling rules for each Item individual.

For improved dependency management, seed configurations are organized as graphs. That is, developers can implement dependencies amongst resources, enabling the loading of data based on previously collected individuals. Taking advantage of the wide variety of existing life sciences web services, we can find direct or transitive connections amongst almost all kinds of data types, from genomics to pharmacology. This allows for the creation of advanced data integration workflows, combining multiple Concept individuals and enabling the aggregation of millions of triples in the seed’s knowledge base.

COEUS future developments include the addition of a user-friendly GUI to configure new seeds. In the meanwhile, and considering the setup files OWL/RDF nature, relying on Protégé is advisable to ease the configuration process. In this widely used ontology-modelling tool, the configuration can be written, tested and visually organized.

### Resource integration

#### Extract-transform-load

Several issues arise with the integration of data from distributed resources. Traditional warehousing techniques revolve around advanced algorithms for extracting data from a specific source, transforming it into the warehouse model and replicating the data in the new integrated dataset. In COEUS, this Extract-Transform-Load data warehousing process is specialized for a semantic web environment, enhancing the inward data flow from CSV or XML files, and from SQL or SPARQL query results to sets of triple statements.

The initial problem that arises when building new knowledge bases relates to data access and to the diversity of formats involved in the data import process. This can be easily overcome through the use of standardized URIs. Whether we are accessing a REST web service or a MySQL database, most programming technologies allow configuring this access through a simple URI. For instance, a JDBC connection to a MySQL database is specified as *jdbc:mysql://<host>:<port>/<dbname>?user=<dbuser>&password=<dbuserpwd>* and access to UniProt’s protein data is made through *http:// http://www.uniprot.org/uniprot/<proteinid>.<format >*. The similarities are clear and enable a simplification of the external resources’ configuration: all resources will have a URI property and query variables, for example.

Along with data format and location diversity, the heterogeneity of each distributed data model further increases the complexity associated with COEUS data integration process. We know from the start that data will come through in all sorts of formats and models. To overcome these situations, COEUS adds an intermediate abstraction layer between the external resources and the internal knowledge base.

The goal behind this abstraction layer is to convert the data being integrated to a general model-independent format. In practice, the implemented method generates a network for each new item, mapping the configured predicates to the values from the external resources. With this data abstraction, the triplification process can take place, enabling the generation of triple statements from the abstracted data model for further storage in COEUS knowledge base.

This complex strategy required the construction of purpose-specific wrappers. These methods access external resources and process data, using connectors, based on a set of configuration properties, the selectors.

Selectors are property sets defining the data location in a specific resource and what predicate will be added to the knowledge base during the integration process. Connectors control these particular data mappings: independent and generic modules to load information from external resources in CSV and XML file formats, or from SQL or SPARQL query results (one for each Resource specialization in COEUS model). They possess a common set of configuration properties defining the data type, where the data are located, the relationships to existing data, and other module-specific definitions. This information is stored in the seed configuration files. For instance, the XML module configuration must include the original data source address and a collection of selector properties, XPath queries, which will be performed against the read XML, corresponding to the data being mapped. Likewise, selectors are SQL query results names, CSV column numbers and SPARQL query variables, in their respective connectors.

**Figure 3:**
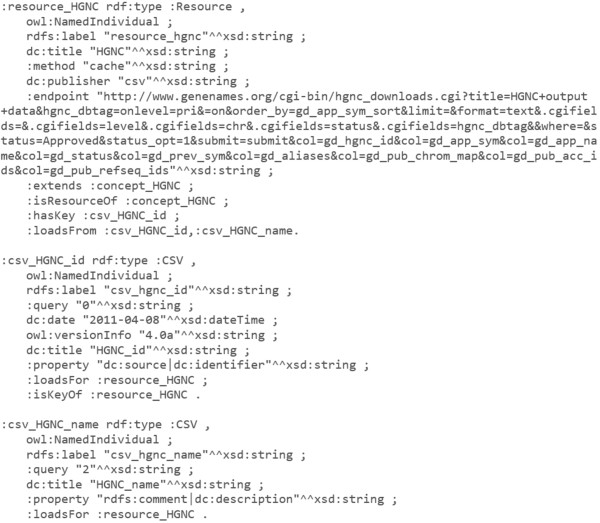
**Simplified HGNC resource configuration sample.** Three resource individuals are used to configure a simple resource loading from HGNC gene list into a new seed’s knowledge base. The “resource_HGNC” is configured to load data from the selected :endpoint object, send it for processing using the CSV connector, property dc:publisher, and map the results from two :CSV individuals, property :loadsFrom. In these, the :query predicate defines which column object will map to the predicates listed in :property. Hence, in the “csv_HGNC_id” individual, data obtained from column 0 (zero) of the HGNC CSV file, property :query, will be mapped into two triple statements with the same subject, the dc:source and dc:identifier predicates and with the same object, dynamically read from the CSV source.

Continuing in our proteomics scenario, Figure 3 details an integration configuration sample to load known human genes data into a COEUS seed. This list is maintained by the HUGO Gene Nomenclature Committee (http://www.genenames.org/), which provides a REST service for getting the gene list in CSV format. The model for this seed comprises a Resource individual that configures how the connectors load this list in our seed, populating a “HGNC” Concept under the “Gene” Entity.

The data loading process uses connectors to initiate a data triplification process. Data are enriched through the dynamic generation of new triples based on specified configuration properties. With this semantic web-based abstraction we are augmenting the scope of data in one-dimensional CSV files or bi-dimensional SQL tables to a multi-dimensional triplestore. Again, the advantages surrounding semantic web’s expressivity become apparent, liberating existing data into new richer formats and enabling innovative knowledge reasoning and inference features.

The richness of this triplification process resides in the connector’s flexibility. The selectors within a given connector allow us to match any content into our semantic graph using a primary key for the subject, any property mapped from the seed ontology as predicate, and selection results as objects. These are then used to generate each new Item map on the fly, which is then converted into a set of statements and inserted into the knowledge base.

The triplification process is also highlighted in Figure 2 (1). Data from XML and CSV files, or SQL query results does not possess any related semantics (only column names or objects). Hence, explicit descriptions for the new predicates need to be set up. Since with COEUS we can map data in XML and CSV files or SQL query results to any predicate in any ontology and to more than one predicate at once, we can expand the meaning of non-semantic data by explicitly declaring it as the object of a specific statement. For example, transforming a “name” column from a table into the object of a foaf:name or rdfs:label predicate improves the underlying meaning of collected data as it is now a part of a normalized ontology with a standardized thesaurus. In spite of SPARQL queries’ results already having some implicit semantics, the selector can match data into new predicates or the original predicate. In this way, COEUS can replicate triples or expand them to richer entities.

#### Integration strategies

Data integration in COEUS can be performed using three distinct approaches according to the seed needs or to how data are provided by each service. These methods – *cache*, *complete* and *map* – are defined by the :method property in a resource configuration and are short summary follows.

COEUS can replicate triples or expand them to richer entitiesThe *cache* method is used by default. This method enables standard data loadings from external resources, generating new items and triplifying data directly. This method is used to load new data from scratch, as shown in the HGNC example detailed in Figure 3.

COEUS can replicate triples or expand them to richer entitiesThe *complete* method appends new triples to items already in the seed triplestore. A sample example involves adding data regarding the HGNC-approved gene symbol to complete individual item information for the knowledge base configured in Figure 3.

COEUS can replicate triples or expand them to richer entitiesThe *map* integration method enables the creation of custom direct connections amongst individuals. This means that we can infer entire sets of new mappings, object property associations, amongst items after the data are loaded. Considering the example from Figure 3, with a list mapping UniProt protein identifiers with their HGNC gene counterparts we can establish custom links between proteins and genes already in the knowledge base.

Both the *map* and *complete* integration methodologies use the :extends configuration predicate from COEUS’ internal ontology to define the concept whose individual item list will be enriched.

With COEUS’ triplification strategy the data integration process is abstracted from the data itself. With room for one or more ontologies in each seed, new unique relationships can be established, disregarding the traditional constraints we are used to finding in legacy software. Data are collected and connected using distinct methods and miscellaneous import formats. This is ideal for optimizing all kinds of new data-powered applications, namely in the life sciences field, where heterogeneous data models with limited relationships are common.

### Exploring collected data

With COEUS we tackle the lack of outward interoperability in existing life sciences information systems. Drawbacks such as poor web service availability, complex and closed data models, or vendor-specific formats are common in bioinformatics. To overcome these limitations, COEUS includes an extensive API providing interoperable services, and leverages on semantic web’s openness to deliver advanced knowledge discovery, exploration, reasoning and inference features. This API is organized in two sections – *internal* and *external* – detailed next.

#### Internal API

COEUS’ *internal* API includes two interfaces for accessing the seed’s knowledge base: a Java API and a JavaScript library. The Java API is an abstraction over Jena’s internal methods, providing a more direct way of accessing COEUS data structures. Hence, actions to access items, concepts or entities, or to add new statements are more straightforward. This API is ideal when developing seeds using the Java programming language. This allows developers to fully explore Java’s features in the creation of new web-based and semantic-powered information systems.

COEUS also includes a JavaScript library (available under *…/assets/js/sparqler.js*) that enables direct connections to each seed’s SPARQL endpoint. With this, it is possible to query and process data from the knowledge base with the powerful SPARQL query language. Queries are built and executed with JavaScript, allowing the retrieval of data as easily handled JSON objects. This library further increases rapid application deployment with COEUS. Combining the JavaScript development framework’s facilities with a streamlined JavaScript-based data retrieval access results in huge potential for the creation of semantic web applications with outstanding user interfaces.

#### External API

The *external* API comprises three layers enabling access to data in the seed: REST services, a SPARQL endpoint and LinkedData views. With these modules, developers can create client applications in any programming language and exchange content in multiple formats (CSV, JSON, RDF/XML or HTML).

COEUS’ RESTful services API provides a set of methods to access data in the knowledge base through simple GET requests. In addition to more complex operations, there is a straightforward service to request basic triple sets. This service enables the building of custom statements with specific subject, predicate or object properties. The *…/api/triple/<sub>/<pred>/<obj>/<format>* URL can be composed with any valid values, enabling fast and iterative access to an instance’s entire knowledge base. Another COEUS’ key feature is the SPARQL endpoint. With an open SPARQL endpoint, available by default at *./sparql*, all stakeholders have full access to a seed’s knowledge base, enabling complex queries and more insightful data retrieval operations. A form for querying each seed triplestore is available at http://bioinformatics.ua.pt/coeus/sparqler/. This form allows developers to test their SPARQL queries before including them in their own applications.

A clear advantage for using the SPARQL endpoint or REST services is access to data in multiple formats. Whereas requesting data in JSON format is optimal for lightweight web application development, it might be necessary to import data in CSV format into an Excel sheet or transform XML content into a new structure. This variety further increases COEUS’ overall flexibility, improving its use in modern application platform environments. To define the desired result format from SPARQL queries, we just need to provide the “output” query string parameter. Similarly, with the REST service, the final query string argument defines the output format. Through its multiple subdivisions, the LinkedData guidelines [17] empower a completely interoperable knowledge ecosystem, where resources are directly accessible through their URIs and their semantic descriptions establish meaningful connections to other miscellaneous data types.

With the LinkedData interface COEUS completes the interoperability features required to enhance modern service composition ecosystems. This ability to make integrated and enriched data available in the linked open cloud without complex configuration tasks or tricky deployment processes is a defining feature of COEUS, taking it one step further in semantic web for life sciences innovation.

#### Usage example

A trivial case study can be setup to test the various elements composing COEUS’ APIs. For this matter, knowledge regarding the *breast cancer type 2 susceptibility* protein (UniProt accession number P51587) will be collected from COEUS sample dataset. These results are obtained from the graph of relationships where a representation of this individual, mapped in COEUS sample knowledge base as :uniprot_P51587, is an active subject. The methods for accessing these data are detailed next.

**Figure 4:**
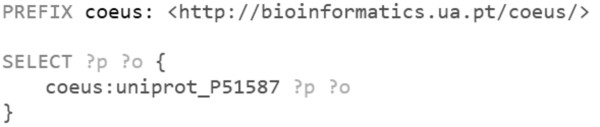
**SPARQL endpoint API query example.** Trivial SPARQL query to obtain data from the set of triple statements where the coeus:uniprot_P51587 is the subject.

 • Java. To obtain these data in Java, the *getTriple()* API method must be invoked, defining what filter to use and the desired XML output format: *pt.ua.bioinformatics. API.getTriple(“coeus:uniprot_P51587”, ”p”, ”o”, “xml”);*

 • REST. The desired protein data can be obtained, in CSV format for example, through a direct GET request to the public REST interface at http://bioinformatics.ua.pt/coeus/api/triple/coeus:uniprot_P51587/p/o/csv.

 • SPARQL. UniProt P51587 data can be queried from COEUS SPARQL endpoint, available at http://bioinformatics.ua.pt/coeus/sparql, with the query highlighted in Figure 4.

 • LinkedData. The requested protein data can be explored through a LinkedData browser pointed to http://bioinformatics.ua.pt/coeus/resource/uniprot_P51587. Additionally, the same address provides a summary view for regular web browsers.

Examples and full documentation can be found online in the documentation (http://bioinformatics.ua.pt/coeus/documentation/).

#### Knowledge federation

COEUS enables a virtual distributed knowledge network, the aforementioned knowledge garden. With multiple seeds in place, performing queries across the various COEUS instances and inferring results on the fly opens up immense data integration and interoperability possibilities. A case study for COEUS’ federation support regards the answers for the following scientific question: *What are the PDB identifiers for the protein structures and the MeSH term identifiers associated with the human BRCA2 gene?*

**Figure 5:**
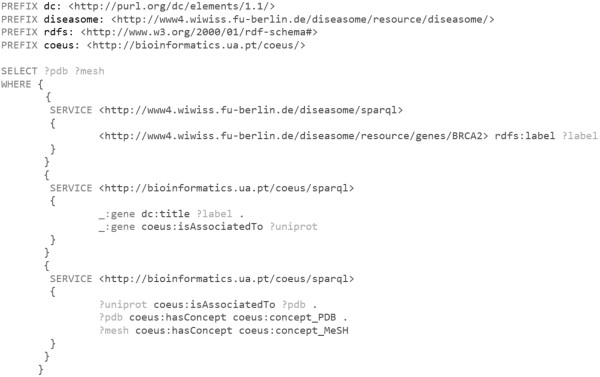
**SPARQL distributed query example.** SPARQL query exploring the distribution features made possible by using the SERVICE keyword to define independent endpoints. This SPARQL query will cross data from multiple knowledge bases to obtain a list of PDB identifiers and MeSH term identifier associated with the given gene. COEUS’ default SPARQL endpoint is replicated to virtually simulate the query distribution.

To answer the proposed question, the federated query shown in Figure 5 links three distinct services, i.e. SPARQL endpoints. The query is processed in real time through the SPARQL endpoint, with the following steps:

1. The Diseasome SPARQL endpoint is queried to obtain the label for the human BRCA2 gene (?label).

2. The ?label variable is passed to the first COEUS seed, acting as the selection clause for the gene and enabling access to a set of UniProt proteins associated with it (?uniprot).

3. The ?uniprot variable is shared with the third SPARQL endpoint, where data regarding PDB identifiers (?pdb) and MeSH term identifiers (?mesh) are selected.

## Results and discussion

COEUS semantic web application framework is suitable for use in the most diverse scenarios. The first use case implementation of COEUS revolves around the rare diseases research field, although its use can be explored in many other areas.

### Case study

Extracting relationships between rare diseases and specific genotype features is the subject of major research projects such as the Human Variome Project [18], the 1000 Genomes Project [19] or the European GEN2PHEN Project [20]. Once we can explore the core potential of genetics, proteomics, pharmacology or clinical databases, we will be able to grasp the meaning and impact of sequence mutations in human health.

Diseasecard is a web portal targeted at clinicians and geneticists, focusing on supplementing rare disease information with connections to genotype-to-phenotype resources [21]. However, in Diseasecard’s previous version, collected data was static, locked and unusable outside the system, which makes this system an excellent case study for COEUS. A new semantic Diseasecard seed is available at http://bioinformatics.ua.pt/dc4/. For example, the *breast cancer* phenotype workspace is accessible at http://bioinformatics.ua.pt/dc4/entry/114480.

**Figure 6:**
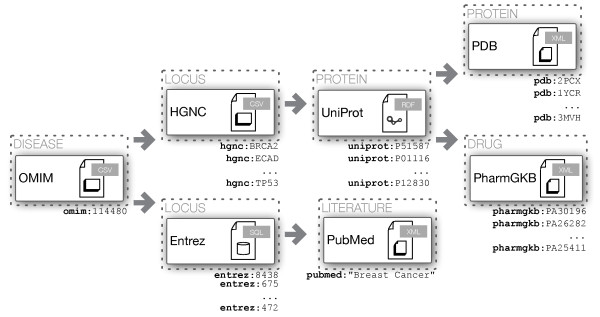
**Subset of Diseasecard’s integration graph.** This seed takes advantage of COEUS’s flexible integration engine to acquire data from heterogeneous and distributed CSV, XML, SQL and SPARQL resources. The integration process generates a rich data network. For example, starting with Breast cancer in OMIM (114480) we obtain multiple genes from HGNC database (BRCA2, TP53…), which are used individually next to obtain a list of UniProt identifiers (P51587, P12830…). From UniProt data we also extract PharmGKB (PA30196, PA26282…) and PDB (2PCX, 1YCR…) identifiers, among others. This process continues until data is fully integrated for all resources in Diseasecard’s configuration.

Figure 6 displays an overview of Diseasecard’s seed configuration sub-graph, highlighting some of the integrated resources and their data types. Initially the system is populated with data from OMIM’s morbid map, available by request at http://omim.org/downloads. CSV connectors and selectors are used to configure this data import using the “cache” method. This file also contains information regarding gene-disease associations (from OMIM to HGNC). Hence, individuals for HGNC Item are also created. This triggers the need for a new connector to complete HGNC individuals’ data, as shown in the “Resource Integration” section. Using the gene identifier we obtain protein associations from UniProt and using its RDF service we fill in missing protein data (http://www.uniprot.org/uniprot/P51587.rdf contains info about Protein P51587, for instance). Furthermore, UniProt’s individual protein set is a rich data source, which we also use to “cache” PDB, PROSITE and InterPro items. OMIM identifiers are mapped to NCBI Entrez entries (using GeNS [22]), which are respectively mapped to PubMed literature pointers. A PubMed Resource individual configures how to “complete” literature details from NCBI XML web services. With this strategy, COEUS permits the straightforward integration of more than 10 distinct data types in Diseasecard, resulting in the deployment of a new web information system without complex data acquisition operations.

The new Diseasecard’s ontology file is available at http://bioinformatics.ua.pt/dc4/diseasecard.owl and the used COEUS setup file at http://bioinformatics.ua.pt/dc4/dc4_setup.rdf.

With COEUS, Diseasecard’s data are no longer closed. Anyone can enrich new or existing software by taking advantage of the included API to get data from Diseasecard. For instance, to get a JavaScript object with all OMIM rare diseases collected in Diseasecard, developers just need to issue a GET to http://bioinformatics.ua.pt/dc4/api/triple/sub/coeus:hasConcept/coeus:concept_OMIM/js. Likewise, basic disease information such as name, location, phenotype, associated diseases or items can be obtained in XML format at http://bioinformatics.ua.pt/dc4/api/triple/coeus:omim_114480/pred/obj/xml.

Launching Diseasecard is the first step towards a COEUS-powered interoperable open data ecosystem. Further seeds can be easily deployed in the future, focusing on a variety of niche fields and targeting distinct stakeholders. Next, taking advantage of COEUS’ knowledge federation features, we can holistically reason over genotype-to-phenotype knowledge and infer new meaningful connections between our genes and rare diseases.

### Evaluation

Modern research and healthcare scenarios require an improved life sciences technical infrastructure to deal with a multitude of data sources, each generating input data for various kinds of stakeholders, from policy makers to wet-lab scientists. This is where semantic web technological advantages come into play. Being part of a comprehensive semantic knowledge network is engaging everyone involved in life sciences research. Whilst awareness of this new development paradigm is spreading, the scarcity of streamlined and easy to use semantic web application frameworks is delaying the much-needed update.

Moreover, developers must adapt their applications to semantic web environments as soon as possible. COEUS empowers life sciences application developers with a streamlined semantic web deployment tool. Using COEUS results in a smoother transition from monolithic applications with relational or flat-file back ends to an information system sustained by a fully semantic software stack. While its flexibility makes it an indirect competitor to other semantic frameworks in the life sciences ecosystem, it also makes it more suitable to solve a wider range of bioinformatics problems, namely of knowledge integration, interoperability and federation.

Common semantic web migration strategies emphasize the development of translation languages enabling the mapping from relational connections to the semantic web graph. On the one hand, basic languages simply map tables and columns to a new model following the proposed ontology. On the other hand, more innovative systems already permit the extension of existing data connections, enriching their meaning and expressiveness. This approach is further divided in two topics: some mappings are dedicated to forming new semantic web triple sets from existing data, whereas others enable access to relational databases as virtual RDF graphs.

The latter languages are complemented with translation applications, using the newly mapped model to provide a semantic data version. Triplify [23], Virtuoso (http://virtuoso.openlinksw.com/) and D2R server [24] have managed to employ these new techniques. Instances with DailyMed, Diseasome, DrugBank, and SIDER data were created using D2R, providing useful SPARQL data integration endpoints.

Despite these remarkable advances in mapping technology, there is still a lack of data insertion and triplification features, which are more challenging tasks being backed by large-scale research projects. In a sense, this is the shortest route to publishing existing datasets in modern semantic web formats. However, COEUS is used to produce full semantic systems, whereas these systems only work with mappings from relational databases to a semantic web version. Therefore, the resulting semantic view over relational data is poorer than what can be achieved with COEUS and its comprehensive data integration connectors.

Bio2RDF [25] or DBPedia [26] collect a vast amount of data in outsized triplestores. With the same decision-support goals as traditional warehouse systems, these platforms adopt advanced extract-transform-load techniques to triplify existing data into a semantic format, storing it in high-performance triple stores.

Bio2RDF’s biology environment enables it to be a remarkable life sciences semantic database, collecting data pointers from a wide variety of domains, from genes to proteins or from pathways to publications, for instance. Despite the natural quality and appeal of these large semantic data repositories [27], they do not fit common niche fields. Whilst Bio2RDF diversity and size will expand its usage to the level of systems like UniProt or BioMART, these features also make it unsuitable for smaller and restricted environments such as specific gene, disease or model organism information systems [28]. Even when new software integrates connections to Bio2RDF data, the system’s core will be composed of small datasets and other precise information bits gathered from external databases or wet-lab file systems.

S3DB proposes a new data management model for integrating biomedical knowledge capable of helping in miscellaneous niche environments [29]. S3DB provides developers with tools to construct their own ad-hoc semantic web applications instead of beginning the development with an empty box. The proposed solutions for managing ontologies or locked data repositories make it suitable for closed environments. However, data integration and software interoperability features are still very primitive. In the life sciences, providing mechanisms for importing or translating data from various formats into the knowledge base and to enable the sharing of information amongst ontologies is imperative [30].

COEUS extract-transform-load warehouse integration strategy does not produce triplification results as extensive as Bio2RDF or DBPedia, as it is built to generate new information systems from scratch, simplifying the translation process. Nonetheless, the ability to integrate data from CSV and XML files, or from SQL and SPARQL query results, matching loaded content with any ontology, in real time, makes COEUS’ data integration connectors suitable for acquiring data from the most widely used data sources and web services. Hence, while it is possible to replicate the entire Bio2RDF dataset using COEUS, it is more appropriate to deploy smaller focused instances and connect their data with the native federation layer.

COEUS’ semantic storage performance also falls short in comparison to tools such as OWLIM, and the Jena-based engine underperforms when compared to more complete systems like Virtuoso, as shown by miscellaneous benchmarks [31-33]. Nevertheless, with COEUS we provide a complete semantic web application software stack in a single package. Hence, the “semantic web in a box” approach, delivering the minimum set of tools to deploy semantic web applications without relying on the installation of additional software.

COEUS’s carefully produced and sharing-prone framework model allows deeper software interoperability along with data integration. To have fully interoperable applications we must establish connections from and to other systems. If the external resource interaction model is scalable and flexible enough, we are one step closer to providing a means for software interactions with our system. With COEUS we simplify these efforts: by using SPARQL queries to load data and previously designed ontologies for modeling, the difficulty shifts from “how” the systems works to “what” the system contains.

In addition to the SPARQL endpoint, COEUS’ API also includes REST services and LinkedData views for the exploration of acquired and triplified data. With these data exchange options, COEUS facilitates outward interoperability with existing or new software, allowing for interactions across multiple systems. Furthermore, the combination of COEUS’ interoperability features in several framework instances activates a distributed knowledge federation layer [34]. Using SPARQL, applications may perform queries over more than one endpoint, triggering a holistic view over a large-scale COEUS-powered ecosystem.

In summary, COEUS combines software features in the realm of semantic web mappings, data integration and triplification, warehousing and services interoperability in a “one size fits all” package.

## Conclusions

Enhanced biomedical semantics are the cornerstone for a better understanding of our human condition. Life sciences’ demands are typically one step ahead of computer science developments. About a decade past completion of the human genome, the extent of data generated in biomedical settings is growing vertiginously and goes beyond what current common computational systems can handle. While it will not solve all problems in bioinformatics, the semantic web emerges as the most viable alternative to lead the next-generation of biomedical software, capable of tackling the majority of life sciences’ knowledge-related challenges.

Despite being targeted at life sciences developers, COEUS can be used in various real world settings. Whether we are dealing with the corporate domain or with a specific topic management, data are generated in large quantities and with complex innate relationships. While we do not envisage COEUS replacing infrastructures already set up in these areas, the framework is suitable for the quick deployment of new *ad hoc* knowledge bases anywhere.

COEUS is a new semantic web framework comprising the skeleton for creating best-of-breed semantic web information systems, supported by four key features. The comprehensive exploration ontology streamlines the transition from primitive data environments into a new semantic knowledge base. The flexible integration engine enables an easy triplification of data from distributed and heterogeneous data sources. The extensive API makes all data collected in a COEUS seed interoperable through a rich set of services. At last, with multiple COEUS instances in place, a turnkey solution for semantic knowledge federation is delivered. As a result, the COEUS framework is a pioneering system, with an innovative “semantic web in a box” approach that greatly improves the modern application deployment workflow.

## Availability and requirements

**Project name:** COEUS

**Project homepage:**http://bioinformatics.ua.pt/coeus

**Operating system(s):** Platform independent

**Programming language:** Java

**Other requirements:** Java 1.6 or higher, Apache Tomcat 6.0 or higher, MySQL 5.0 or higher

**License:** Creative Commons Attribution-NonCommercial-ShareAlike 3.0

**Any restrictions to use by non-academics:** none

## Competing interests

The authors declare no competing interests on the presented work.

## Authors’ contributions

PL conceived, researched and developed the COEUS framework under JLO supervision and coordination. All authors read and approved the final manuscript.
